# On Gun-Shot Wounds

**Published:** 1813-11

**Authors:** 


*For the Medical and Physical Journal.*

ON GUN-SHOT WOUNDS.

THE following treatise is a translation from the surgical work of M. Richerand, a distinguished physiologist and professor of the Parisian school of medicine. To this translation some slight additions have been made; and one or two paragraphs on extracting instruments have been omitted, for the purpose of substituting an account of the instruments of M. Chevalier, which seem to be the most useful. The observations of M. Richerand merit peculiar attention, because, in addition to his acquaintance with the experience of the French army surgeons, it is evident he is not ignorant of the opinions of English surgeons, and above all those of Mr. John Hunter. To the latter circumstance, almost almost as much as to the former, may, perhaps, be attributed the abandonment of those officious intermeddlings with the processes of nature, which characterised the practice of the French, so late as at the commencement of the French revolution. At that time, it would have appeared unpardonable negligence to leave a gun-shot wound without a certain number of *incisions* and *unbridlings*. "The first indication of the systematic surgeon," says M. Percy,* "is to *change the nature* of this wound, and convert it, as much as possible, into a bleeding wound." We shall see M. Richerand restricting the application of this practice, and pointing out, with considerable discrimination, though not in the most systematic manner, the cases in which *incisions* and *unbridlings* ought to be employed.

Gun-shot wounds are characterised by the disorganization of their surface. The extreme contusion observed in these wounds depends on the rapidity with which the body producing them is moved. The parts it touches are converted into a blackish slough, which caused the ancients to think that bodies, projected by gunpowder, were heated, and produced actual burns. Reason and experience have taught us, that, however great may be the rapidity of a projectile, it does not acquire any sensible heat during its course. The degree of heat which would render a ball capable of burning the human body, would be sufficient to melt the ball.

The wounds of fire arms do not bleed unless a large vessel should be opened: the neighbouring parts are livid, and the shock which accompanies is so sudden and violent, that the part struck experiences a kind of stupor, of which the whole animal economy frequently partakes.

The history and the treatment of gun-shot wounds was filled with the most pernicious errors, before Ambrose Paré had established the true theory of them. Cannon balls and bullets sometimes produce the most serious injuries, without hurting the skin. The soft parts have been reduced to a kind of jelly, and the bones themselves broken, although the integuments were uninjured; and this effect was long attributed to the displacement of the air by the projectiles. It was believed that this elastic fluid, when suddenly displaced by the shock of a cannon ball, was capable of pressing surrounding bodies with sufficient violence to tear their substance; but how can we conceive such a pressure in the midst of the free air? The effect observed ought to be produced constantly, when a ball passes near; but we may every

* Chirurgien d'Armée, p. 181, Ed. 1792.

day day see bullets carry away the plume, the hat, the clothes, and even the hair of the soldier, without his experiencing any other inconvenience.

*The oblique action* of balls on the human body easily explains this extreme contusion, in which the skin is not affected. Sometimes also it depends on the weakness with which these balls strike, when, having expended the greater part of the motion impressed on them, they act only by virtue of their weight. They are then designated by the name of spent balls.

When the contusions of fire arms are pretty violent, the muscles and cellular membrane bruised, and reduced to a kind of jelly, similar to the lees of wine, the bone sometimes broken, the limb is often in a state of torpor, which inevitably brings on gangrene.

Gun-shot wounds may have only one, or they may have two outlets, the ball being arrested more or less deeply in the thickness of the part, or having passed quite through it. In the last case the two openings are diametrically opposite, in the greater number of instances: pretty often, however, the outlet does not exactly correspond with the entrance of the ball, its direction having been changed by the resistance opposed to it by a bone, a cartilage, a tendon, or even an aponeurosis. Thus a bullet, having pierced the skin of the leg near the internal ankle, has been known to glide between the tibia and the skin, ascend, and come out near the knee; sometimes having struck the forehead, it has issued at the temple, etc. Books relate a great many instances of these singular deviations. The directness of a ball's course will be in proportion to the rapidity of its motion, and also to the softness of the parts it passes through.

The wounds of musquet balls generally have the form of the body which produces them: they are round, square, or oblong; but when they have two openings, that at the entrance is always larger than that at the exit of the ball. Its edges are depressed; there is a depression near the entrance, while the parts are, as it were, raised, and make a projection towards the other opening. This difference takes place, because, at the moment the ball meets the limb, it strikes it with its full force, which it loses when it is buried in the thickness of the parts, in overcoming their resistance. The skin at the entrance is supported by the whole thickness of the limb; this point of support favors the solution of continuity, and prevents laceration: the contusion is also, for the same reasons, greater at the entrance of the ball, and when the swelling, which is always proportionate to the contusion, has taken place, the difference between the two openings is more strongly marked, for the entrance is more swelled than the outlet. These explanations are so well founded, that Le Dran, speaking of wounds of the head, remarks, there is no difference between the entrance and outlet, because the point of support is of the same nature.

The livid yellow color of the parts around a gun-shot wound, arises from the ecchymosis produced by the violence with which the blood is driven back, the slough preventing the escape of the humors. They swell the part, and much increase the importance of the wound. The part struck is benumbed, weighty, and in this state of torpor defends itself feebly against the entrance of liquids; the organic activity being almost extinguished, gangrene comes on, and makes the most rapid advances. This state of stupor and insensibility is especially fatal when the whole body experiences it; and this takes place in consequence of severe commotions, as when the body is struck by a large ball, or any other body of a certain bulk. In this state died the light horseman of whom Quesnay speaks: the state of stupefaction was such, that when they proposed amputation, the man answered "it was none of his business."

Jaundice is seen to occur suddenly from gun-shot wounds; also shiverings, faintings, and other nervous accidents, which caused the old surgeons to believe that gunpowder carried a secret poison into wounds; but the attrition which organs undergo, and the violent shock, of which the whole system partakes in a greater or less degree, are sufficient to explain the most mischievous consequences.

The complication arising from foreign bodies concealed in the wound, often exists in this species of injuries. These bodies are, either the ball itself, or portions of the clothes, which it has carried into the flesh. When the wound has but one opening, it very probably contains a foreign body: this is not certain, however; a number of cases are related, in which the ball, after making a deep wound of some inches, has been found in the shirt of the wounded man. In these cases, the linen is not even torn, but merely driven into the wound.

When this has two openings, we may conjecture that the ball has passed out; but even then, fragments of the clothes may remain in the passage, and this is the more easy, because these bodies being lighter, and pushed with a less force than the ball, are not capable of passing through an equal space.

Thus then the first indication in gun-shot wounds is to proceed to the search of foreign bodies, with which it may be complicated. Nothing can contra-indicate this search, but but the danger of causing an unmanageable hemorrhage, by detaching the slough. A variety of ball forceps have been invented either to discover or extract the balls and other foreign substances, which the wound may contain. The finger is preferable to them all, when it is capable of reaching the foreign body; for the resistance made by bones and tendons, which lie in the course of the wound, may easily pass for some extraneous body.

The best forceps are, perhaps, those recommended by Mr. Chevalier. They are of two kinds; one for the extraction of balls, which is nearly like straight tooth forceps, except that the swell near the extremity is more considerable, for the purpose of receiving the ball. The other forceps are formed like common dressing forceps, but are of a greater size. A silver instrument has been invented, the form of which somewhat resembles that of an unblown tulip, the leaves of which are contrived to expand after the instrument has reached the ball, and thus having received it into their cavity, to close and retain it.*

It is useful, in searching for foreign bodies, with which gun-shot wounds may be complicated, to cause the patient to be placed in the situation he was when he received the wound. By means of this precaution, and carefully feeling the parts about the wound, Ambrose Paré discovered, in the Marshal Brisac, a ball placed between the shoulder blade and the spine, which had escaped the search of many surgeons, who had employed the probe alone.

It must not be forgotten that balls sometimes deviate from a straight course, in a very singular manner. In order that they should undergo these deviations, it is not necessary they should meet with bone, cartilage, or tendon. The mere difference of medium may produce a kind of refraction; for since water is able to turn a ball from a right line, the soft parts of the body will produce this effect in a greater degree, as their density is greater than that of a simple liquid.

These deviations of balls may cause us to believe that they have penetrated a cavity whose surface only they have passed over. Such, no doubt, was the case of a young drummer of the Swiss guards, who received a wound in the shoulder on the 10th of August. The ball had struck under the clavicle, and passed to the inferior angle of the scapula. Professor

* This is not a modern invention. We find in Dionis the figure of an instrument of very similar construction, which he calls *Alphonsin* from the name of the inventor.

Boyer

Boyer extracted it by making a counter-opening. How could it pass through the upper part of the breast without wounding any of the important organs it must meet in its passage? Besides, it presented asperities, which evidently proved it had rubbed against osseous parts.

Sometimes, the nicest examination is not successful in discovering them; their passage is so tortuous, and they are so much refracted by the hard parts they meet in the way, that it is impossible to get at them. Then, by carefully examining the parts about the wound, and especially the part diametrically opposed to it, we perceive a foreign body through a greater or less thickness of parts, which it is necessary to incise, in order to extract it. We determine to make these counter openings the more readily, because a ball never can be extracted by the passage it had entered, without a considerable and painful traction on the parts. The new opening also facilitates the discharge of pus, and singularly shortens the cure, which is rendered tedious by the lodgment of this fluid, when the wound has only one outlet.

We must spare neither time nor pains for the extraction of foreign bodies, for their presence is a continual cause of irritation: they aggravate the accidents of wounds, and make them degenerate into fistulas. Sometimes, however, balls lie closely lodged in bones, during a long series of years, without danger and without pain. In some cases they pass a long way under the skin, making their way through the cellular membrane, without bringing on inflammation. The passage is then usually marked by the appearance of a reddish line in the skin. Balls have been seen to become a cause of irritation after they had been a long time quietly lodged in the body, bringing on a suppuration which removed them. We should not be too obstinate in searching for extraneous substances, because a very long and painful search might cause worse accidents than the matters lodged in the wound. When they are very difficult to find, we must abandon their expulsion to nature, or at least wait for symptoms to determine our course.

Is it always necessary to enlarge gun-shot wounds by incising their orifices? Some authors have laid down as a general rule the dilatation of these wounds. This operation, say they, besides that it singularly facilitates the search for foreign bodies, prevents the strangulation of the parts, when their inflammatory swelling comes on. More timid practitioners have gone so far as to forbid dilatation in all cases. According to them, these incisions increase the local disorder, facilitate gangrene, and are not without danger to the nerves, the the vessels, and the tendons, which may be implicated in them. It is true that this practice has been carried to excess; but the proscription of its abuse ought not to be extended to its use: it is, therefore, indispensable to determine the cases in which it may be necessary.

(To be continued.)

